# Intramolecular Transformations of 3-Cyanoamino- and 3-Cyanoimino-1,2-diferrocenylcyclopropenes

**DOI:** 10.3390/molecules14093161

**Published:** 2009-08-26

**Authors:** Elena Ivanovna Klimova, Tatiana Klimova, Marcos Flores-Alamo, Leon Vladimirovich Backinowsky, Marcos Martinez Garcia

**Affiliations:** 1 Universidad Nacional Autónoma de México, Facultad de Química, Cd. Universitaria, Coyoacán,C.P. 04510, México D.F., Mexico; E-mails: klimova@servidor.unam.mx (T.K.), mfa24s99@gmail.com (M.F-A.); 2 N.D. Zelinsky Institute of Organic Chemistry , Russian Academy of Sciences, 47 Leninsky prosp., 117913 Moscow, Russia; E-mail: leon_backinowsky@mail.ru (L.B.); 3 Instituto de Química, Universidad Nacional Autónoma de México, Cd. Universitaria, Coyoacán, C.P.04510, México D.F., Mexico; E-mail: margar@servidor.unam.mx (M.M.G.)

**Keywords:** diferrocenylcyclopropenylium salts, cyanoamino(diferrocenyl)cyclopropenes, [amino(cyanoimino)methyl]-1,2-diferrocenylethenes, 3-amino-6-ferrocenyl-5-ferrocenyl-methyl-1,2,4-triazine

## Abstract

3-Cyanoamino-1,2- and -2,3-diferrocenylcyclopropenes **6a,b** and **11a,b** prepared by the reaction of diferrocenylcyclopropenylium salts with sodium cyanamide undergo smooth intramolecular transformations with both conservation of the three-membered ring [affording 3-cyanoimino-1,2-diferrocenylcyclopropene (**8**)] and its opening [affording *Z*-3-morpholino- and *Z*-3-piperidino-3-(cyanoimino)-1,2-diferrocenylprop-1-enes **7a**,**b** and *Z-*3-cyanoimino-2,3-diferrocenyl-1-methylthioprop-1-ene(**10**)]. 3-Cyano-imino-1,2-diferrocenylcyclopropene (**8**) reacts with hydrazine to form 3-amino-6-ferrocenyl-5-ferrocenylmethyl-1,2,4-triazine (**12**) and *Z*-2,3-diferrocenylacrylohydrazide *N*-cyanoimide (**13**) as a result of intramolecular transformations. The structures of the compounds obtained were determined by IR, ^1^H- and ^13^C-NMR spectroscopy and mass spectrometry. The structures of compounds **7a** and **10** were additionally confirmed by their X-ray diffraction analysis data.

## Introduction

**Scheme 1:**
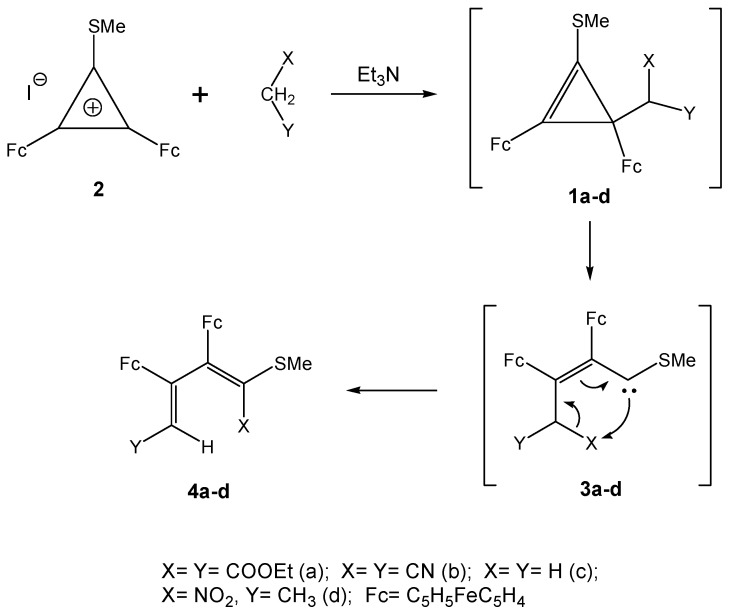
Reaction of diferrocenyl(methylthio)cyclopropenylium iodide (**2**) with active methylene reagents.

The range of natural compounds comprising cyclopropane or cyclopropene fragments is fairly broad. Many of them are of particular interest due to their peculiar inherent biological activities [1,2,3,4,5]. In synthetic practice, compounds with a three-membered ring represent both the target products and intermediates in various carbon skeleton transformations [6,7,8]. These processes include, as a rule, ring opening reactions [8] into intermediate allylic cations or vinylcarbenes that serve as “building blocks” in organic synthesis. The presence of ferrocenyl substituents in the three-membered ring greatly facilitates these ring opening reactions [9,10,11,12,13]. This allows the use of ferrocenylcyclopropanes/cyclo-propenes prepared by directed synthesis for their subsequent transformation into long-chain conjugated systems [6,8] and carbo- and heterocycles [14,15,16,17] incorporating iron-containing fragments. The effect of the nature of other functional groups and hetero-substituents on the ease of the three-membered ring opening of ferrocenylcyclopropenes has been but scantily explored. In particular, it has been established that the small ring opening occurs very readily for 2,3-diferrocenyl-1-methylthiocyclo-propenes **1a-d** [15,16,17,18,19]. These are formed in the reaction of diferrocenyl(methylthio)cyclopropenylium iodide (**2**) with active methylene reagents (diethyl malonate, malononitrile, nitroalkanes) and are further converted *via* 2,3-diferrocenyl-1-methylthiovinylcarbenes **3a-d** into diene systems **4a-d** with ferrocenyl substituents and terminal functionalities as a result of intramolecular migration of a functional group (Scheme 1).

Studies on this type of chemical transformations are of undoubted interest for specialists in theoretical, physical and synthetic organic chemistry, as well as to the search for compounds with such valuable properties. In the present work, we report the results of studies on the reactions of sodium cyanamide with diferrocenyl(morpholino)- and -(piperidino)cyclopropenylium tetrafluoroborates **5a,b** and diferrocenyl(methylthio)cyclopropenylium iodide (**2**).

## Results and Discussion

**Figure 1:**
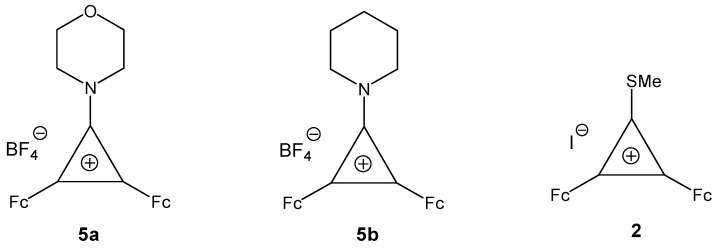
Starting diferrocenylcyclopropenylium salts **5a, 5b**, and **2**.

The starting diferrocenylcyclopropenylium salts **5a, 5b**, and **2** (Figure 1) were prepared from 2,3-diferrocenyl-cyclopropenone as described earlier [16,20,21].

**Scheme 2:**
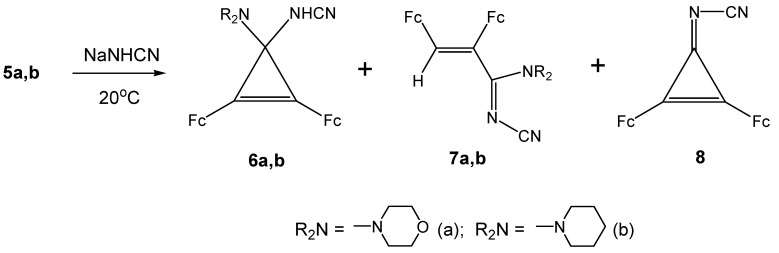
Syntheses of **6a,b**, **7a,b** and **8**.

We found that diferrocenyl(morpholino)- and -(piperidino)cyclopropenylium tetrafluoroborates **5a,b**) react regioselectively with sodium cyanamide at 20 ºC (Scheme 2) to yield the following reaction products, *viz.*, **6a,b**, **7a,b**, and **8** in the ratio ~ 1:3:2 (see Experimental section).

These compounds were separated by column chromatography on alumina. Eluted first were cyclopropenes **6a** (**6b**). In solid state, they are orange powders that gradually decompose on storage. Their structures were established based on the data from ^1^H- and ^13^C-NMR spectroscopy and mass spectrometry. Thus, the corresponding ^1^H- and ^13^C^-^NMR spectra contain the necessary number of signals for the protons and carbon atoms corresponding to the methylene groups of the morpholine and piperidine substituents and to two ferrocenyl fragments. The ^13^C-NMR spectra also contain the signals for the nitrile groups (**6a**, δ 120.56 ppm; **6b**, δ 121.75 ppm), and the ^1^H-NMR spectra contain signals at δ = 5.37 and 5.52 ppm, respectively, typical of the -NH group.

**Figure 2:**
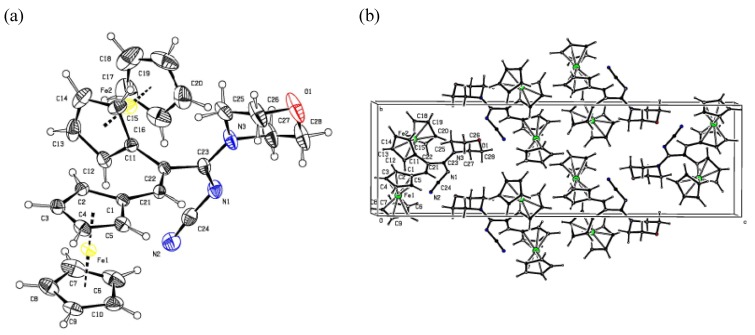
(a) Crystal structure of **7a**; (b) Crystal packing of **7a**.

Eluted next from the column were compounds **7a** and **7b** as single isomers, judging from their ^1^H- NMR spectra. Their structures were established by ^1^H- and ^13^C-NMR, IR, and UV spectroscopy. The ^1^H-NMR spectra of compounds **7a** and **7b** contain, in addition to the signals for the protons and carbon atoms corresponding to the methylene groups of the morpholine and piperidine substituents and to two ferrocenyl fragments, one singlet each for low-field protons at δ 6.45 ppm (**7a**) and δ 6.39 ppm (**7b**), which allowed assigning them tentative structures of 3-morpholino-3-(cyanoimino)- and 3-piperidino-3-(cyanoimino)-1,2-diferrocenylprop-1-enes **7a** and **7b**, respectively. The structure of compound **7a** followed also from X-ray diffraction analysis of a single crystal prepared by crystallization from dichloromethane [22], which proved its structure as *Z*-3-morpholino-3-(cyanoimino)-1,2-diferrocenyl-prop-1-ene. The general view of the molecule **7a** is shown in Figure 2a, the packing of molecules in a crystal is shown in Figure 2b, and the main geometrical parameters are given in Table 1. Data from the X-ray analysis show that the N=C bond in the azadiene is somewhat longer [*d* = 1.314(3) Å] than the standard value of 1.29 Å [23,24]. The lengths of C-Fe and C-C bonds in the ferrocenyl substituents are close to the standard values [25]. By analogy, the structure of *Z*-3-piperidino-3-(cyanoimino)-1,2-diferrocenylprop-1-ene was ascribed to compound **7b**.

**Scheme 3:**
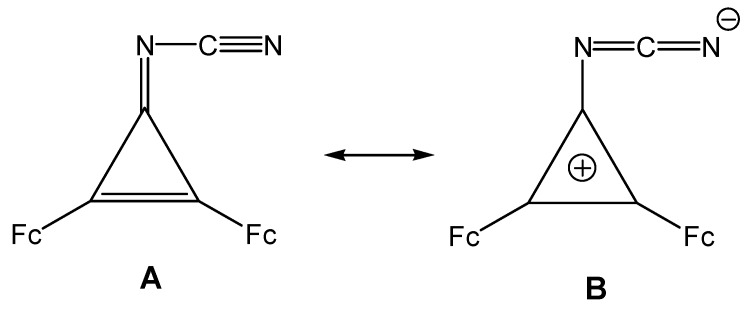
Pseudoaromatic character of 3-cyanoimino-1,2-diferrocenylcyclopropene **8**.

Eluted last from the chromatographic column was 3-cyanoimino-1,2-diferrocenylcyclopropene (**8**). It is possibly the pseudoaromatic character of these structures (A↔B) (Scheme 3) that determines this order of elution.

**Table 1:** Selected bond lengths and bond angles for compounds **7a** and **10**.

Selected bond lengths (Å)		Selected bond angles (^o^)	
**7a**			
C(24)-N(2)	1.151(4)	N(2)-C(24)-N(1)	172.6(3)
C(24)-N(1)	1.329(4)	C(23)-N(1)-C(24)	119.3(2)
C(23)-N(1)	1.314(3)	N(1)-C(23)-N(3)	117.5(2)
C(23)-C(22)	1.495(3)	N(1)-C(23)-C(22)	123.2(2)
C(22)-C(21)	1.334(3)	C(23)-C(22)-C(21)	118.8(2)
C(23)-N(3)	1.332(3)	C(22)-C(23)-N(3)	119.3(2)
N(3)-C(25)	1.454(4)	C(21)-C(22)-C(11)	123.0(2)
C(1)-C(21)	1.462(3)	C(23)-N(3)-C(27)	121.4(3)
**10**			
N(1)-C(25)	1.338(6)	C(21)-N(1)-C(25)	120.1(4)
N(2)-C(25)	1.145(6)	N(2)-C(25)-N(1)	172.1(5)
C(21)-N(1)	1.303(5)	N(1)-C(21)-C(22)	122.7(3)
C(21)-C(22)	1.499(5)	C(21)-C(22)-C(23)	115.8(3)
C(22)-C(23)	1.350(5)	C(22)-C(23)-S(1)	126.9(3)
C(23)-S(1)	1.739(4)	C(23)-S(1)-C(24)	100.6(2)
S(1)-C(24)	1.789(5)	N(1)-C(21)-C(1)	118.5(4)
C(22)-C(11)	1.459(5)	C(1)-C(21)-C(22)	118.8(3)
C(1)-C(21)	1.442(5)	C(11)-C(22)-C(23)	127.8(3)

The cationic part of this structure is cyclopropenylium with the Hückel aromaticity [26,27], which makes the contribution of structure B quite important [7]. Spectroscopic characteristics of cyclopropene **8** corroborate its structure.

**Scheme 4:**
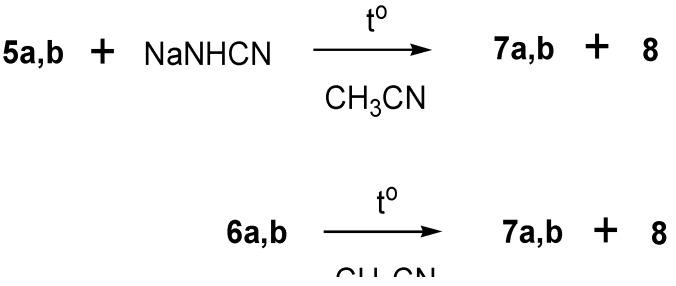
Synthesis of **7a, 7b** and **8**.

We also found that the reactions of diferrocenylcyclopropenylium salts **5a** (**5b**) with sodium cyanamide carried out in boiling acetonitrile (10-12 h) afforded compounds **7a** (**7b**) and **8**. The same products were formed upon prolonged boiling of cyclopropenes **6a** (**6b**) in acetonitrile (Scheme 4).

**Scheme 5:**
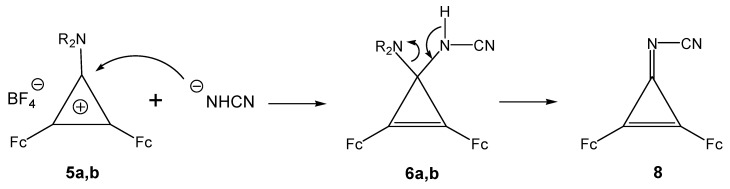
Plausible mechanism of the formation of **6a**, **6b** and **8**.

It thus follows that azadienes **7a (7b**) and cyanoiminocyclopropene **8** result from transformations of tetrasubstituted diferrocenylcyclopropenes **6a** (**6b**). A plausible mechanism of the reaction includes initial nucleophilic attack of the cyanamide anion on the C-1 atom of the three-membered ring of cyclopropenylium cations **5a** (**5b**) with formation of 3-cyanoamino-1,2-diferrocenyl-3-morpholino- (or -3-piperidino)cyclopropenes **6a** (**6b**) (Scheme 5).

**Scheme 6:**
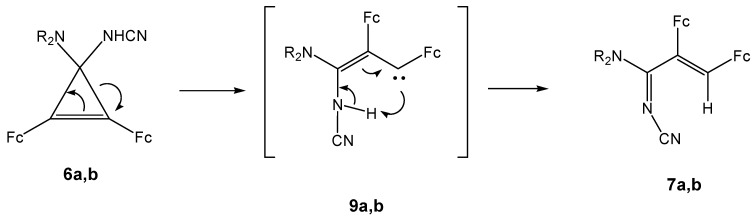
Plausible mechanism of the formation of **7a** and **7b.**

Subsequent intramolecular transformation of tetrasubstituted cyclopropenes **6a** (**6b**) with elimination of a molecule of morpholine (piperidine) (Scheme 6) affords cyanoiminocyclopropene **8**. Compounds **6a** (**6b**) undergo also three-membered ring opening [16,17,18,19] giving cyanoamino-diferrocenyl(morpholino)- or [-(piperidino)]vinylcarbenes **9a** (**9b**), which are stabilized as a result of proton migration (Scheme 6).

**Scheme 7:**
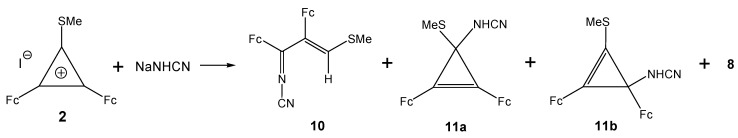
Synthesis of **10, 11a** and **11b.**

Unlike cyclopropenylium salts **5a** (**5b**), diferrocenyl(methylthio)cyclopropenylium iodide (**2**) reacts with sodium cyanamide at 20 ºC to yield mainly two products, **10** and **8,** and small amounts of cyclopropenes **11a** and **11b** (Scheme 7).

**Figure 3:**
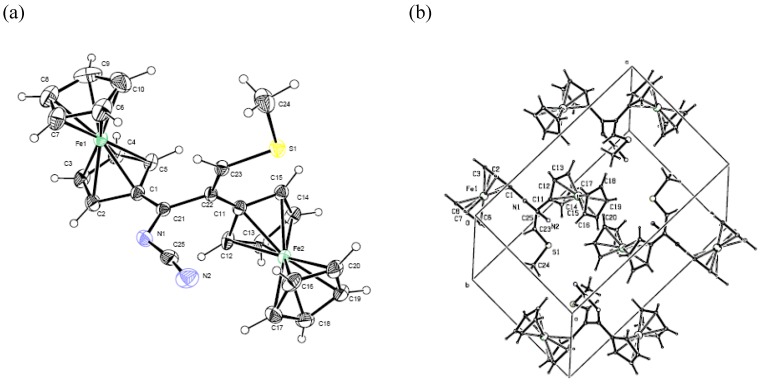
(a) Crystal structure of **10**; (b) Crystal packing of **10.**

The physicochemical characteristics of compound **8** were identical to those of the product prepared from diferrocenyl(morpholino)- and -(piperidino)cyclopropenylium salts **5a** and **5b**. The structure of compound **10** was established based on the data from IR, UV, ^1^H- and ^13^C-NMR spectroscopy and mass spectrometry. The structure of compound **10** was also confirmed by X-ray diffraction analysis of a single crystal prepared by crystallization from chloroform [22]. The perspective view of the molecule **10** is shown in Figure 3a, the crystal packing diagram is shown in Figure 3b, and selected bond lengths and bond angles are listed in Table 1.

According to the data from X-ray analysis, compound **10** is *Z-*3-cyanoimino-2,3-diferrocenyl-1-methylthioprop-1-ene. The length of the N=C bond in compound **10** [*d* = 1.303(5) Å] is somewhat longer than the standard value of 1.29 Å [23,24].

In our opinion, the fact that the N=C bond in compounds **7a** and **10** is longer than the standard value of 1.29 Å is due to the presence of a conjugated system of double bonds in these compounds. In addition, it can be observed from Table 1 that σ-bonds in these compounds are somewhat shorter than the corresponding standard values. We think that the latter observation is also due to the presence of the conjugated system of bonds. 

Isomeric 3-cyanoamino(diferrocenyl)cyclopropenes **11a** and **11b** (yields ~10 and 6%, respectively) are unstable oily products that undergo rapid decomposition on storage under ordinary conditions. Their structures were established based on the data from IR, ^1^H- and ^13^C-NMR spectroscopy and mass spectrometry. Structures **11a** and **11b** were assigned to the isomers of methylthiocyclopropenes based on the position of the proton signals of the substituted cyclopentadiene rings in the ^1^H NMR spectra. In cyclopropene **11a**, all signals for the protons of the C_5_H_4_ fragments are present in a lower field than the singlets of the protons of unsubstituted cyclopentadienyl groups. In cyclopropene **11b**, the signals for the protons of one of the C_5_H_4_ fragments of the ferrocenyl substituent are upfield relative to the signals for the protons of the C_5_H_5_ group, which corresponds to the effect of electron-donating MeS-C=C-Fc fragment of the cyclopropene.

**Scheme 8:**
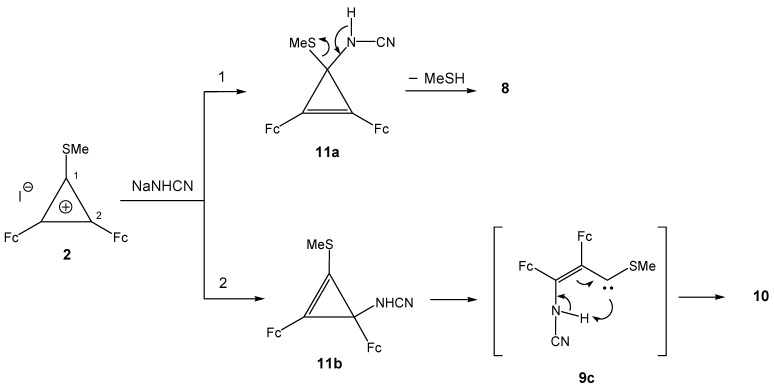
Plausible mechanism of the formation of **8** and **10**.

Obviously, one of the reaction products of iodide **2** with sodium cyanamide, *viz*., cyanoiminocyclopropene **8**, results from intramolecular transformation of cyclopropene **11a** (Scheme 8) analogous to that of 3-cyanoamino-1,2-diferrocenyl-3-morpholino- ( or -3-piperidino)cyclopropenes **6a** (**6b**) (see Scheme 5). The other reaction product, compound **10**, is formed upon three-membered ring opening [16,17,18,19] in 3-cyanoamino-2,3-diferrocenyl-1-methylthiocyclopropene **11b** to vinylcarbene **9c**, whose stabilization owing to the proton transfer to the carbine center affords azadiene **10** (Scheme 8).

The results obtained demonstrate different effects of the heterosubstituents on the regioselectivities of reactions of morpholino- (or piperidino-) and methylthio-diferrocenylcyclopropenylium salts with the cyanamide anion and on relative stabilities, *i.e.*, proneness to opening of their three-membered rings. The reaction products of salts **5a,b** are formed exclusively upon the attack of the cyanamide anion on the C-1 atom of the cyclopropenylium ring. Such a regioselectivity is uncharacteristic of the reaction of the 1-methylthio- analog; at the same time, transformations of tetrasubstituted cyclopropene intermediates **11a** and **11b** occur much more smoothly.

**Scheme 9:**
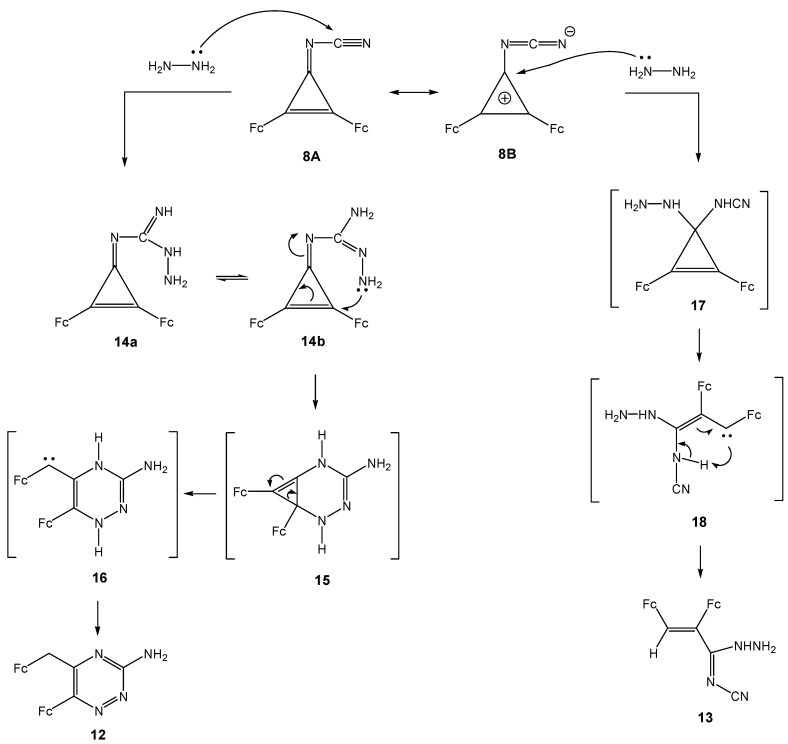
Plausible mechanism of reaction of 3-cyanoimino-1,2-diferrocenyl-cyclopropene **8** with hydrazine.

Further, we observed that 3-cyanoimino-1,2-diferrocenylcyclopropene (**8**) as a pseudoaromatic compound reacts with hydrazine in boiling ethanol to give two reaction products, *viz*., compounds **12** and **13** (Scheme 9). The nucleophilic attack of the hydrazine nitrogen atom on the carbon atom of the nitrile group results in 3-amino-6-ferrocenyl-5-ferrocenylmethyl-1,2,4-triazine (**12**) *via* tentative intermediates **14**, **15**, and **16**. The structure of compound **12** was established by IR, ^1^H- and ^13^C-NMR spectroscopic and mass spectrometric data. Thus the IR spectrum of compound **12** contains absorption bands of a free NH_2_ group (ν 3487 cm^-1^) and ferrocenyl substituents. The ^1^H-NMR spectrum contains signals for protons of two ferrocene fragments, a singlet of an FcCH_2_ group (δ 4.32 ppm) and a broad singlet of protons of the NH_2_ group (δ 6.94 ppm). Data from the ^13^C-NMR spectrum corroborate the structure of compound **12**.

The nucleophilic attack of hydrazine on the C-1 atom of the three-membered ring in **8B** affords product **13** resulting from opening of the small ring in intermediate **17** to vinylcarbene **18** and its subsequent intramolecular transformation; the structure of the final product **13** followed from the spectroscopic data.

## Experimental

### General

All the solvents were dried according to the standard procedures and were freshly distilled before use [28]. Column chromatography was carried out on alumina (Brockmann activity III). The ^1^H- and ^13^C-NMR spectra were recorded on a Unity Inova Varian spectrometer (at 300 and 75 MHz, respectively) for solutions in CDCl_3_, with Me_4_Si as the internal standard; chemical shifts δ are given in ppm. The IR spectra were measured on a Perkin Elmer FT-IR spectrophotometer (Spectrum RXI) using KBr pellets. The mass spectra were obtained on a Varian MAT CH-6 instrument (EI MS, 70 eV). Elementar Analysensysteme LECO CHNS-900 was used for elemental analyses. The unit cell parameters and the X-ray diffraction intensities were recorded on a Siemens P4 diffractometer. The structures of compounds **7a** and **10** were solved by the direct method (SHELXS -97 [29]) and refined using full-matrix least-squares on F^2^.

### Synthesis of diferrocenylcyclopropenylium salts (**5a, 5b**, and **2**)

Diferrocenylcyclopropenylium salts **5a, 5b**, and **2** were prepared from 2,3-diferrocenylcyclopropenone as described earlier [16,20,21]: 2,3-diferrocenylcyclopropenone was obtained from the ferrocene and tetrachlorocyclopropene in the presence of AlCl_3_ according to the standard procedure [20]; Ethoxy(diferrocenyl)cyclopropenylium tetrafluoroborate was obtained from 2,3-diferrocenylcyclopropenone in the presence of triethyl-oxonium tetrafluoroborate (1.0 M solution in dichloromethane) [21]; Morpholino- and piperidino-(differocenyl)cyclopropenylium tetrafluoroborates were obtained from ethoxy(diferrocenyl)-cyclopropenylium tetrafluoroborate and morpholine or piperidine in dichloromethane [21]; 3-Diferrocenylcyclopropenethione was obtained by treating ethanolic differocenyl(morpholino)-cyclopropenylium tetrafluoroborate with an aqueous solution of NaSH [16]; 2,3-Diferrocenyl-(methylthio)cyclopropenylium iodide (**2**) was obtained from the 2,3-diferrocenylcyclopropenethione and iodomethane [16]. Freshly prepared and thoroughly dried tetrafluoroborates **5a,b** and iodide **2** were employed in the reactions with sodium hydrogencyanamide. Reactions were carried out in freshly distilled dry solvents.

### Reaction of dialkylamino(diferrocenyl)cyclopropenylium tetrafluoroborates with sodium hydrogencyanamide

Sodium hydrogencyanamide (0.64 g, 10 mmol) was added to a solution of 1-amino-2,3-diferrocenylpropenylium tetrafluoroborate **5a,** **b** (5 mmol) in dichloromethane (chloroform, acetone, or acetonitrile) (100 mL), and the mixture was stirred in a dry inert atmosphere at ~20 °C (~24-36 h) or under reflux (14-20 h). The solvents were removed *in vacuo*, and the residues were chromatographed on alumina (hexane-dichloromethane, 4:1) to give compounds **6a**, **b**, **7a**, **b** and **8**.

*3-Cyanoamino-1,2-diferrocenyl-3-morpholinocyclopropene* (**6a**): Yield 0.32 g (12%); red-violet powder; mp 174-175 °C; ^1^H-NMR: δ 3.16 (m, 4H, 2CH_2_), 3.56 (m, 4H, 2CH_2_), 4.09 (s, 5H, C_5_H_5_), 4.24 (s, 5H, C_5_H_5_), 4.05 (m, 2H, C_5_H_4_), 4.15 (m, 1H, C_5_H_4_), 4.43 (m, 1H, C_5_H_4_), 4.68 (m, 2H, C_5_H_4_), 5.01 (m, 2H, C_5_H_4_), 5.37 (bs, 1H, NH); ^13^C-NMR: δ 61.23 (C), 65.21 (2CH_2_), 66.34 (2CH_2_), 69.24, 70.43 (2C_5_H_5_), 67.93, 68.05, 69.04, 69.37, 70.82, 71.10, 72.34, 72.47 (2C_5_H_4_), 80.22, 81.23 (2C*_ipso_*Fc), 120.56 (CN), 139.11 (2C); MS: *m/z* 533 [M]^+^; Anal. Calcd. for C_28_H_27_Fe_2_N_3_O: C, 63.07; H, 5.10; Fe, 20.95; N, 7.88; Found: C, 62.91; H, 5.17; Fe, 21.06; N, 7.69.

*Z*-*3-morpholino-3-(cyanoimino)-1,2-diferrocenylprop-1-ene* (**7a**): Yield 1.01 g (37%); violet crystals; mp 229-230 °C; λ_max_ (CHCl_3_, 20^o^C): 207.31, 207.80, 235.05, 235.55 nm; IR (KBr): 473, 483, 541, 723, 773, 824, 861, 898, 921, 930, 977, 1000, 1024, 1052, 1105, 1115, 1214, 1259, 1286, 1324, 1352, 1383, 1411, 1440, 1484, 1536, 1626, 2181, 2851, 2977, 3082 cm^-1^;^1^H-NMR: δ 3.48-3.92 (m, 8H, 4CH_2_), 4.27 (s, 5H, C_5_H_5_), 4.29 (s, 5H, C_5_H_5_), 4.04 (m, 1H, C_5_H_4_), 4.22 (m, 1H, C_5_H_4_), 4.25 (m, 1H, C_5_H_4_), 4.28 (m, 1H, C_5_H_4_), 4.32 (m, 1H, C_5_H_4_), 4.34 (m, 1H, C_5_H_4_), 4.41 (m, 1H, C_5_H_4_), 4.82 (m, 1H, C_5_H_4_), 6.45 (s, 1H, CH=); ^13^C-NMR: δ 66.22 (2CH_2_), 66.52 (2CH_2_), 69.53, 69.74 (2C_5_H_5_), 67.98, 68.15, 68.88, 68.95, 69.12, 70.01, 70.25, 71.29 (2C_5_H_4_), 78.18, 80.28 (2C*_ipso_*Fc), 126.07 (CN), 133.89 (CH=), 144.27 (C), 169.78 (C=N); MS: *m/z* 533 [M]^+^; Anal. Calcd. for C_28_H_27_Fe_2_N_3_O: C, 63.07; H, 5.10; Fe, 20.95; N, 7.88; Found: C, 63.19; H, 4.98; Fe, 20.87; N, 7.99.

*3-Cyanoimino-1,2-diferrocenylcyclopropene* (**8**): Yield 0.56 g (25%); orange crystals; mp 214-216 °C; IR (KBr): 472, 483, 540, 551, 558, 722, 772, 824, 861, 898, 920, 930, 977, 1000, 1024, 1052, 1104, 1115, 1214, 1258, 1286, 1323, 1352, 1381, 1411, 1439, 1494, 1534, 1626, 1864, 2179, 2850, 2892, 2977, 3082 cm^-1^; ^1^H-NMR: δ 4.28 (s, 10H, 2C_5_H_5_), 4.71 (m, 4H, C_5_H_4_), 4.93 (m, 4H, C_5_H_4_); ^13^C-NMR: δ 70.48 (2C_5_H_5_), 72.65, 73.31, 73.35, 73.38 (2C_5_H_4_), 88.36, 88.64 (2C*_ipso_*Fc), 121.28 (CN), 132.64 (C), 145.51 (C=N); MS: *m/z* 446 [M]^+^; Anal. Calcd. for C_24_H_18_Fe_2_N_2_: C, 64.62; H, 4.06; Fe, 25.04; N, 6.28; Found: C, 64.51; H, 4.12; Fe, 24.89; N, 6.19.

*3-Cyanoamino-1,2-diferrocenyl-3-piperidinocyclopropene* (**6b**): Yield 0.38 g (14%); red-violet powder; mp 172-173 °C; ^1^H-NMR: δ 1.58 (m, 2H, CH_2_), 1.74 (m, 4H, 2CH_2_), 2.99-3.06 (m, 4H, 2CH_2_), 4.05 (s, 5H, C_5_H_5_), 4.21 (s, 5H, C_5_H_5_), 3.99 (m, 1H, C_5_H_4_), 4.03 (m, 2H, C_5_H_4_), 4.55 (m, 1H, C_5_H_4_), 4.63 (m, 1H, C_5_H_4_), 4.71 (m, 2H, C_5_H_4_), 5.10 (m, 1H, C_5_H_4_), 5.52 (bs, 1H, NH); ^13^C-NMR: δ 23.95 (CH_2_), 25.64 (2CH_2_), 50.31 (2CH_2_), 58.19 (C), 69.31, 70.52 (2C_5_H_5_), 68.04, 68.12, 69.29, 69.42, 71.02, 72.13, 72.85, 72.90 (2C_5_H_4_), 81.35, 81.41 (2C*_ipso_*Fc), 121.75 (CN), 139.24 (2C); MS: *m/z* 531 [M]^+^; Anal. Calcd. for C_29_H_29_Fe_2_N_3_: C, 65.56; H, 5.50; Fe, 21.03; N, 7.91; Found: C, 65.63; H, 5.38; Fe, 21.15; N, 7.99.

*Z*-*3-Piperidino-3-(cyanoimino)-1,2-diferrocenylprop-1-ene* (**7b**): Yield 1.20 g (45%); violet crystals; mp 195-196 °C; λ_max_ (CHCl_3_, 20^o^C): 205.96, 207.43, 233.82, 237.03 nm; IR (KBr) 472, 481, 540, 722, 773, 822, 860, 896, 920, 930, 978, 1000, 1024, 1050, 1103, 1114, 1213, 1259, 1287, 1321, 1352, 1384, 1412, 1441, 1485, 1536, 1626, 2180, 2852, 2975, 3082 cm^-1^; ^1^H-NMR: δ 1.73-1.92 (m, 6H, 3CH_2_), 3.15-3.72 (m, 4H, 2CH_2_), 4.22 (s, 5H, C_5_H_5_), 4.23 (s, 5H, C_5_H_5_), 4.05 (m, 1H, C_5_H_4_), 4.12 (m, 1H, C_5_H_4_), 4.17 (m, 1H, C_5_H_4_), 4.20 (m, 1H, C_5_H_4_), 4.21 (m, 1H, C_5_H_4_), 4.30 (m, 1H, C_5_H_4_), 4.36 (m, 1H, C_5_H_4_), 4.78 (m, 1H, C_5_H_4_), 6.39 (s, 1H, CH=); ^13^C-NMR: δ 24.05, 25.38, 26.32, 45.19, 49.85 (5CH_2_), 69.42, 69.63 (2C_5_H_5_), 67.92, 68.01, 68.64, 68.99, 69.09, 69.78, 70.62, 70.89 (2C_5_H_4_), 78.40, 80.92 (2C*_ipso_*Fc), 126.07 (CN), 133.0 (CH=), 135.52 (C), 152 46 (C=N); MS: *m/z* 531 [M]^+^; Anal. Calcd. for C_29_H_29_Fe_2_N_3_: C, 65.56; H, 5.50; Fe, 21.03; N, 7.91; Found: C, 65.39; H, 5.61; Fe, 21.18; N, 7.79.

*3-Cyanoimino-1,2-diferrocenylcyclopropene* (**8**): Yield 0.57 g (26%); orange crystals; mp 214-216 °C.

### Reaction of 2,3-diferrocenyl-1-methylthiocyclopropenylium iodide (**2**) with sodium hydrogencyanamide

A solution of compound **2** (2.9 g, 5.0 mmol) in dichloromethane (chloroform, acetone, or acetonitrile) (100 mL) was stirred with sodium hydrogencyanamide (0.64 g, 10 mmol) at ~20 °C (9-12 h) or under reflux for 5 h. Subsequent work-up of the reaction mixtures as described above gave compounds **8**, **10** and **11a,b**.

*3-Cyanoimino-1,2-diferrocenylcyclopropene* (**8**): Yield 0.18 g (8%); orange crystals; mp 215-216 °C.

*Z-*3*-Cyanoimino-2,3-diferrocenyl-1-methylthioprop-1-ene* (**10**): Yield 1.51 g (61%); violet crystals; mp 183-184 °C; λ_max_ (CHCl_3_, 20^o^C): 245.09, 299.36, 299.70, 368 nm; IR (KBr): 474, 495, 540, 613, 677, 723, 774, 818, 829, 866, 889, 1000, 1030, 1048, 1106, 1123, 1216, 1295, 1304, 1338, 1376, 1408, 1432, 1464, 1517, 1567, 1635, 2178, 2919, 3103 cm^-1^; ^1^H-NMR: δ 2.59 (s, 3H, CH_3_), 4.20 (s, 5H, C_5_H_5_), 4.28 (s, 5H, C_5_H_5_), 4.25 (m, 2H, C_5_H_4_), 4.30 (m, 2H, C_5_H_4_), 4.48 (m, 1H, C_5_H_4_), 4.62 (m, 2H, C_5_H_4_), 5.05 (m, 1H, C_5_H_4_), 6.71 (s, 1H, CH=); ^13^C-NMR: δ 18.87 (CH_3_), 69.83, 70.92 (2C_5_H_5_), 68.32, 68.74, 73.27, 73.68 (2C_5_H_4_), 93.22, 99.91 (2C*_ipso_*Fc), 121.15 (CH=), 123.07 (CN), 132.48 (C), 155 91 (C=N); MS: *m/z* 494 [M]^+^; Anal. Calcd. for C_25_H_22_Fe_2_N_2_S: C, 60.36; H, 4.50; Fe, 22.60; N, 5.66; S, 6.43; Found: C, 60.48; H, 4.33; Fe, 22.54; N, 5.72; S, 6.57.

*3-Cyanoamino-1,2-diferrocenyl-3-methylthiocyclopropene* (**11a**): Yield 0.25 g (10%); red-violet powder; mp 163-164 °C; ^1^H-NMR: δ 2.48 (s, 3H, CH_3_), 4.18 (s, 5H, C_5_H_5_), 4.19 (s, 5H, C_5_H_5_), 4.36 (m, 2H, C_5_H_4_), 4.45 (m, 1H, C_5_H_4_), 4.58 (m, 1H, C_5_H_4_), 4.69 (m, 1H, C_5_H_4_), 4.70 (m, 2H, C_5_H_4_), 4.91 (m, 1H, C_5_H_4_), 5.08 (bs, 1H, NH); ^13^C-NMR: δ 16.23 (CH_3_), 58.52 (C), 69.59, 70.13 (2C_5_H_5_), 68.57, 68.86, 69.42, 70.45 (2C_5_H_4_), 85.41, 87.74 (2C*_ipso_*Fc), 122.83 (CN), 126.95, 133.21 (2C); MS: *m/z* 494 [M]^+^; Anal. Calcd. for C_25_H_22_Fe_2_N_2_S: C, 60.36; H, 4.50; Fe, 22.60; N, 5.66; S, 6.43; Found: C, 60.42; H, 4.37; Fe, 22.73; N, 5.47; S, 6.58.

*3-Cyanoamino-2,3-diferrocenyl-1-methylthiocyclopropene* (**11b**): Yield 0.15 g (6%); red-violet powder; mp 158-159 °C; ^1^H-NMR: δ 2.62 (s, 3H, CH_3_), 4.07 (s, 5H, C_5_H_5_), 4.11 (s, 5H, C_5_H_5_), 4.01 (m, 2H, C_5_H_4_), 4.09 (m, 2H, C_5_H_4_), 4.18 (m, 2H, C_5_H_4_), 4.23 (m, 2H, C_5_H_4_), 5.31 (bs, 1H, NH); ^13^C- NMR δ 17.4 (CH_3_), 63.14 (C), 69.46, 69.75 (2C_5_H_5_), 68.41, 68.54, 68.92, 70.04 (2C_5_H_4_), 80.01, 82.91 (2C*_ipso_*Fc), 125.24 (CN), 127.13, 131.84 (2C); MS: *m/z* 494 [M]^+^; Anal. Calcd. for C_25_H_22_Fe_2_N_2_S: C, 60.36; H, 4.50; Fe, 22.60; N, 5.66; S, 6.43; Found: C, 60.21; H, 4.67; Fe, 22.51; N, 5.72; S, 6.35.

### Reaction of 3-cyanimino-1,2-diferrocenylcyclopropene (**8**) with hydrazine

A solution of compound **8** (1.0 mmol) and hydrazine hydrate (2.0 mL) in ethanol (20 mL) was stirred for 6 h at 78 °C. The reaction mixture was evaporated *in vacuo*, and residue was chromatographed (Al_2_O_3_; hexane/ethyl ether, 4:1) to give compounds **12** and **13**.

*3-Amino-6-ferrocenyl-5-ferrocenylmethyl-1,2,4-triazine* (**12**): Yield 0.17g (35%); orange powder; mp 236-238 °C; IR (KBr) 487, 534, 718, 821, 89, 934, 1002, 1038, 1101, 1123, 1171, 1244, 1302, 1360, 1456, 1507, 1586, 1599, 1612, 1651, 2890, 2934, 3091, 3421 cm^-1^; ^1^H-NMR: δ 4.12 (s, 5H, C_5_H_5_), 4.24 (s, 5H, C_5_H_5_), 4.29 (m, 2H, C_5_H_4_), 4.31 (m, 2H, C_5_H_4_), 4.34 (m, 2H, C_5_H_4_), 4.45 (m, 2H, C_5_H_4_), 4.32 (s, 2H, CH_2_), 6.94 (bs, 2H, NH_2_); ^13^C-NMR: δ 57.93 (CH_2_), 69.57, 70.18 (2C_5_H_5_), 68.93, 69.44, 70.34, 70.98 (2C_5_H_4_), 84.88, 90.07 (2C*_ipso_*Fc), 149.13, 152.36, 156.29 (3C); MS: *m/z* 478 [M]^+^; Anal. Calcd. for C_24_H_22_Fe_2_N_4_: C, 60.29; H, 4.64; Fe, 23.36; N, 11.71; Found: C, 60.41; H, 4.53; Fe, 23.51; N, 11.64.

*Z*-*2,3-Diferrocenylacrylohydrazide N-cyanoimide* (**13**): Yield 0.23 g (48%); violet powder; mp 304-305 °C; IR (KBr) 478, 498, 532, 614, 678, 720, 770, 821, 830, 869, 923, 1001, 1027, 1051, 1103, 1120, 1221, 1297, 1302, 1345, 1369, 1411, 1432, 1469, 1523, 1567, 1634, 2172, 2896, 3093, 3165, 3487 cm^-1^; ^1^H-NMR: δ 4.09 (s, 5H, C_5_H_5_), 4.14 (s, 5H, C_5_H_5_), 4.21 (m, 2H, C_5_H_4_), 4.32 (m, 2H, C_5_H_4_), 4.39 (m, 2H, C_5_H_4_), 4.57 (m, 2H, C_5_H_4_), 7.68 (s, 1H, CH=), 8.94 (bs, 3H, NHNH_2_); ^13^C-NMR: δ 69.12, 69.2 (2C_5_H_5_), 67.56, 67.84, 67.96, 68.32, 68.53, 69.02, 69.32, 69.75 (2C_5_H_4_), 86.91, 91.08 (2C*_ipso_*Fc), 125.47 (CN), 134.21 (CH=), 142.08 (C), 158.51 (C=N); MS: *m/z* 478 [M]^+^; Anal. Calcd. for C_24_H_22_Fe_2_N_4_: C, 60.29; H, 4.64; Fe, 23.36; N, 11.71; Found: C, 60.51; H, 4.70; Fe, 23.21; N, 11.79.

### Transformation of 3-dialkylamino-, 3-methylthio-3-cyanamino-1,2-diferrocenylcyclopropenes 6a,b and 11a into 3-cyanimino-1,2-diferrocenylcyclopropene (**8**)

A solution of the compounds **6a, 6b** or **11a** (1 mmol) in ethanol (acetonitrile, benzene) (50 mL) was heated at reflux for 6 h and concentrated. The residue was chromatographed on Al_2_O_3_ (hexane - dichloromethane, 4:1) to give 0.34 - 0.36 g (75 - 81%) (from **6a**), 0.32 - 0.34 g (68 - 76%) (from **6b**) or 0.32 - 0.33 g (71 – 73%) (from **11a**) of compound **8**, mp 214-216 °C.

### Transformation of 3-cyanamino-2,3-diferrocenyl-1-methylthiocyclopropene (**11b**) into Z-3-cyanoimino-2,3-diferrocenyl-1-methylthioprop-1-ene (**10**)

A solution of cyclopropene **11b** (1 mmol) in benzene (50 mL) was heated at reflux for 6 h and concentrated. The residue was chromatographed on Al_2_O_3_ (hexane - dichloromethane, 4:1) to give 0.39 g (79%) of compound **10**, mp 183-184 °C.

## Conclusions

3-Cyanoamino-1,2-diferrocenyl-3-morpholino- (piperidino- or methylthio)cyclopropenes **6a,b, 11a** undergo smooth intramolecular transformations with conservation of the three-membered ring affording 3-cyanoimino-1,2-diferrocenylcyclopropene (**8**). Compounds **6a** and **6b** also undergo three-membered ring opening giving cyanoaminodiferrocenyl(morpholino)- or -(piperidino)vinylcarbenes **9a** (**9b**) which allows the use of 1,2-diferrocenylpropene fragments in the synthesis of diferrocenylhetero-1,3-diene systems **7a** and **7b**. 3-Cyanoamino-2,3-diferrocenyl-1-methylthiocyclopropene (**11b**) is transformed upon three-membered ring opening into Z-3-cyanoimino-2,3-diferrocenyl-1-methylthio-prop-1-ene(**10**). 3-Cyanoimino-1,2-diferrocenylcyclopropene (**8**) reacts with hydrazine to form 3-amino-6-ferrocenyl-5-ferrocenylmethyl-1,2,4-triazine (**12**) and *Z*-2,3-diferrocenylacrylohydrazide-*N*-cyanoimide (**13**) as a result of intramolecular transformations of intermediates **14** and **17** with cyclopropene-ring opening. Thus, the reaction of diferrocenylcyclopropene **8** with hydrazine gives rise to aromatic 1,2,4-trizines with amino substituents in the heterocycle. This novel method of synthesis of 1,2,4-aminotrizines, obviously, requires more detailed studies aimed at extension of its potential for the application in organic synthesis.
